# Nonchylous idiopathic pleural effusion in the newborn

**DOI:** 10.4103/0972-5229.78226

**Published:** 2011

**Authors:** Geeta Gathwala, Jagjit Singh, K. N. Rattan, Kapil Bhalla

**Affiliations:** **From:** Departments of Pediatrics, Pt. B. D. Sharma PGIMS, University of Health Sciences, Rohtak, Haryana, India; 1 Department of Pediatric Surgery, Pt. B. D. Sharma PGIMS, University of Health Sciences, Rohtak, Haryana, India

**Keywords:** Idiopathic neonatal pleural effusion, nonchylous, respiratory distress

## Abstract

Congenital isolated pleural effusion is a rare cause of respiratory distress in neonates. It is usually chylous. Herein, we report a rare case of nonchylous congenital idiopathic pleural effusion.

## Introduction

Congenital isolated pleural effusion is a rare condition with an incidence of about 1 in 12,000 to 1 in 15,000 pregnancies.[1] It is usually chylous.[2,3] Herein, we report a rare case of nonchylous congenital pleural effusion managed successfully.

## Case Report

**Figure 1:**
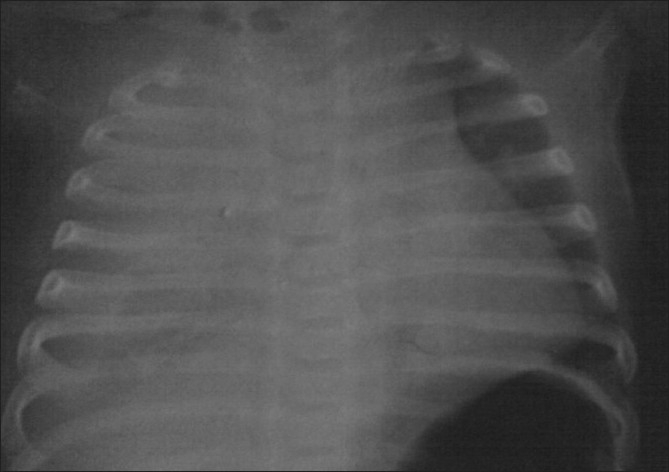
Chest radiograph showing unilateral opacity of right hemithorax and shifting of airway and heart on left side

**Figure 2:**
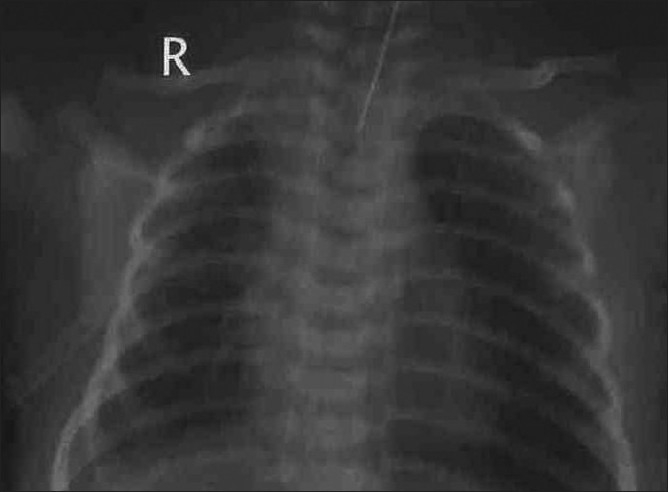
Follow-up chest radiograph showing full expansion of lung fields immediately after chest tube drain

A full-term baby girl, weighing 3 kg, appropriate for gestation, was born by emergency cesarean section done for fetal distress to a 26-year-old unbooked and unsupervised third gravida mother. Baby required resuscitation and the Apgar scores were 4 and 6 at 1 and 5 minutes, respectively. She was in respiratory distress after resuscitation for which she was admitted to the neonatal intensive care unit. Respiratory rate (RR) was 76/minute with subcostal and intercostal recessions and breath sounds were diminished over the right hemithorax. There were no dysmorphic features and the baby was not hydropic. Rest of the systemic examination was normal. An arterial blood gas analysis revealed respiratory acidosis with pH 7.18, HCO_3_18 mmol, PaO_-2_86 mmHg, and PaCO_2_ 54 mmHg. The baby was administrated oxygen via nasal continuous positive airway pressure (CPAP); IV fluids and antibiotics were charted but there was progressive worsening and the baby had to be ventilated [synchronized intermittent mandatory ventilation (SIM V) mode]. An urgent bedside chest X-ray done within the first hour of birth revealed white out of right side with shifting of airway and heart to the left side [Figure 1]. Keeping in mind the possibility of pleural effusion, a diagnostic tap was performed and 50 ml of straw colored fluid was aspirated and chest tube was inserted which drained 500 ml of straw colored fluid. Following pleural drainage, the infant improved remarkably. Repeat X-ray chest showed full expansion of lungs at 24 hours of age [Figure 2], and ultrasonography (USG) chest that was done showed minimal pleural effusion on the right side. Feeding was initiated on day 2. About 40-50 ml of fluid was drained over the next 2 days, which remained clear and nonchylous. Baby was weaned from the ventilator and extubated on day 3. The intercostal drain was removed on day 4. The pleural fluid analysis showed transudate fluid containing protein 1.6 g/l and sugar 86 mg/dl, Cl^−^90 meq/l, triglycerides 10 mg/dl, cholesterol 16 mg/dl, lactate dehydrogenase 110 IU/l, lymphocytes 50/βl; no microorganisms were seen on gram staining and pleural fluid culture was sterile, indicative of nonchylous pleural effusion. The Venereal Disease Research Laboratory (VDRL) test was negative. Computed tomography (CT) of chest was normal. The sepsis screen was normal. USG abdomen and skull were normal and baseline renal and liver function tests were within normal limits. The karyotyping of the infant was normal, i.e. 46, XX. A diagnosis of idiopathic unilateral nonchylous pleural effusion was made. The infant was started on feeds on day 2. She recovered fully with no recurrence of pleural effusion and was discharged on day 14 of life and was doing well on follow-up at 3 months.

## Discussion

Spontaneous or idiopathic neonatal pleural effusion is defined as any effusion in a newborn of age less than 30 days, without any obvious explanation.[4,5] Causes of pleural effusion in the newborn include immune and nonimmune hydrops, Turner and Down syndromes, congenital, pneumonia and wet lung syndrome.[6] The index case had no identifiable cause and was therefore an idiopathic pleural effusion.

Idiopathic neonatal pleural effusion is frequently chylous in nature. Simple effusions are known to turn chylous after establishment of external fat feeds.[4,7] However, in our case, the effusion remained nonchylous even after feeds were initiated. Chylous fluid is milky white in color with triglycerides more than 110 mg/dl (provided there is minimal fat intake), cholesterol between 65 and 220 mg/dl and lymphocytosis with absolute cell count greater than 1000/βl with a lymphocyte fraction greater than 80%.[8] In the index case, the effusion was nonchylous as triglycerides were low (10 mg/dl), the lymphocyte count was low (50/βl) and fluid remained clear despite feeding.

Pleural effusion in the newborn frequently presents with respiratory distress and asphyxia ranging from mild to severe.[7] Early and active resuscitation with intubation and mechanical ventilation are needed to establish chest wall expansion.[9] The index case needed resuscitation at birth and was ventilated.

Effusion presenting antenatally acts as a space-occupying lesion and restricts the development of the fetal lungs, which, as a result, may be hypoplastic. Polyhydramnios may result from interference with normal swallowing because of increased intrathoracic pressure.[10] Polyhydramnios was present in the index case but the lungs were normal.

Management includes thoracocentesis followed by intercostal drain insertion. Antibiotics should be given until an infectious etiology has been excluded.[9,11] Antenatally diagnosed pleural effusions, particularly if present prior to 32 weeks gestation, have a mortality rate as high as 55%.[12,13] Bilateral pleural effusions are frequently associated with pulmonary hypoplasia. Postnatally, effusions persisting for more than 3 days increase the risk of chronic oxygen dependency.[14] In the index case, the pleural effusion resolved by day 3 and the infant did well subsequently.

Respiratory distress remains one of the leading emergencies in neonates. Although idiopathic neonatal pleural effusion remains a rare cause, the awareness of this entity is vital for timely diagnosis and management which can be life saving.
